# Assessment of Normal Sagittal Alignment of the Spine and Pelvis in Children and Adolescents

**DOI:** 10.1155/2013/842624

**Published:** 2013-12-09

**Authors:** Hasan Ghandhari, Hamid Hesarikia, Ebrahim Ameri, Abolfazl Noori

**Affiliations:** ^1^Department of Orthopedic Surgery, Shafa Yahyaeian Hospital, Iran University of Medical Sciences, Tehran, Iran; ^2^Trauma Research Center, Department of Orthopedic Surgery, Baqiyatallah Hospital, Baqiyatallah University of Medical Sciences, Tehran, Iran; ^3^Department of Orthopedic Surgery, Mousavi Hospital, Zanjan University of Medical Science, Zanjan, Iran

## Abstract

*Aim*. We aimed to determine spinopelvic balance in 8–19-year-old-people in order to assess pelvic and spinal parameters in sagittal view. *Methods*. Ninety-eight healthy students aged 8–19 years, who lived in the central parts of Tehran, were assessed. Demographic data, history of present and past diseases, height (cm), and weight (kg) were collected. Each subject was examined by an orthopedic surgeon and spinal radiographs in lateral view were obtained. Eight spinopelvic parameters were measured by 2 orthopedic spine surgeons. *Results*. Ninety-eight subjects, among which 48 were girls (49%) and 50 boys (51%), with a mean age of 13.6 ± 2.9 years (range: 8–19) were evaluated. Mean height and weight of children were 153.6 ± 15.6 cm and 49.9 ± 13.1 kgs, respectively. Mean TK, LL, TT, LT, and PI of subjects were 37.1 ± 9.9°, 39.6 ± 12.4°, 7.08 ± 4.9°, 12.0 ± 5.9°, and 45.37 ± 10.7°, respectively. *Conclusion*. Preoperation planning for spinal fusion surgeries via applying PI seems reasonable. Predicating “abnormal” to lordosis and kyphosis values alone without considering overall sagittal balance is incorrect. Mean of SS and TK in our population is slightly less than that in Caucasians.

## 1. Introduction 

Various parameters have been introduced to describe sagittal alignment of the spine and pelvis. Sagittal spine and spinopelvic parameters are different in adults and children, but these parameters correlate with each other to maintain global balance in both groups. There is no proper description of sagittal spinopelvic balance parameters, characteristics, and relationships in children. Correct concept of normal spinopelvic balance of children would effectively help spinal surgeons assessing spinal deformities and proper planning for treatment. Human standing posture is the result of balance between spine and pelvis [1]. Thoracic kyphosis (TK) and lumbar lordosis (LL) are also in balance with each other in normal standing posture so that the minimal amount of energy is used for maintaining posture [2]. Global sagittal balance must account for the position of the head in relation to the spine and pelvis [3]. The sagittal profile of the spine is usually characterized as being kyphotic between T1 and T12, and lordotic between L1 and L5, but this is not necessarily the case. The differences between normal and pathologic curvatures are less clear in the sagittal plane than in the coronal plane [4–6]. Some studies investigated the amount of normal spinal sagittal curves [7–9] while others evaluated alignment, morphology and pelvic parameters in children [6, 10–13]. Several studies showed the pelvic sagittal morphology affects standing balance in adults especially when LL changes [1, 14, 15]. It has also been proven that pelvic incidence (PI) after adolescence remains relatively constant [10, 14]. TK is one of the main sagittal spinal parameters which show different values in different studies [16], partly due to unclear visualization of T1–T4 vertebrae in lateral spinal radiography [17] and mainly due to various methods of TK measurements; T1–T12 [18], T2–T12 [7], T4–T12 [19], and even T5–T12 [8] have been used to calculate the normal range of TK. There is no consensus on pelvic sagittal geometries in relation to spine in normal children. In addition, abnormal patterns which develop by aging correlate with sagittal curve patterns in childhood [20]. Most papers published in this field have studied white people [19] and to the best of our knowledge there are only few studies on Asians [19, 21–23] and none in Iran. Thus we aimed to determine spinopelvic balance in 8–19-year-old Iranians.

## 2. Methods 

**Figure 1:**
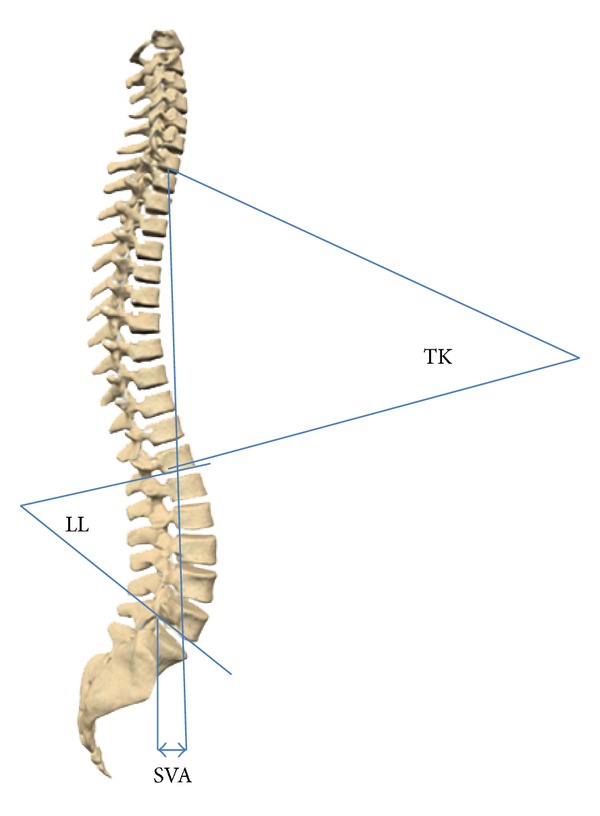
Spinopelvic parameters as measured; TK: thoracic kyphosis, LL: lumbar lordosis, SVA: sagittal vertical axis.

**Figure 2:**
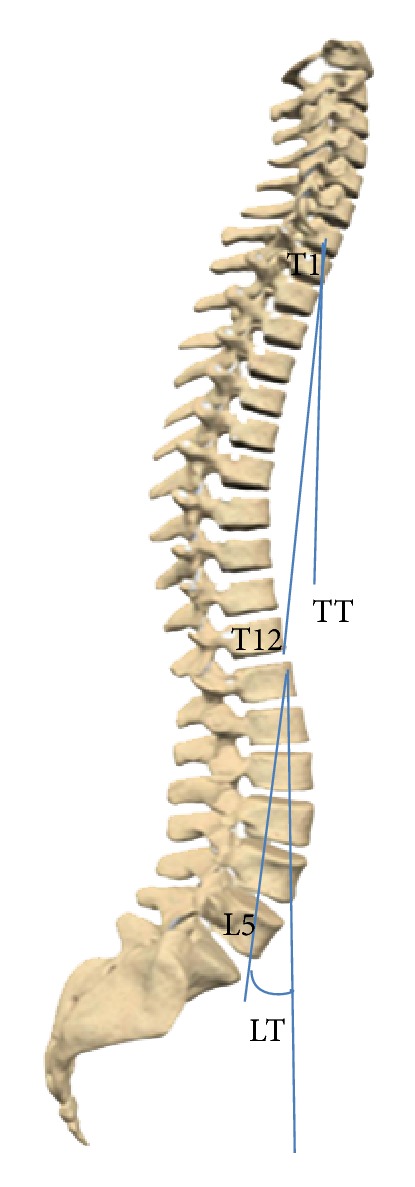
Spinopelvic parameters as measured in the study; TT: thoracic tilt, LT: lumbar tilt.

**Figure 3:**
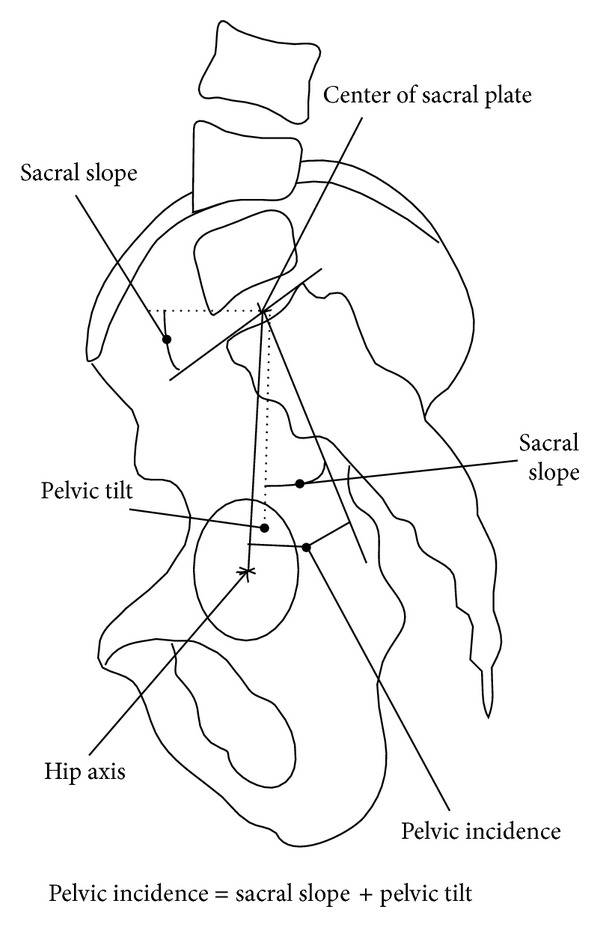
Spinopelvic Parameters as measured in the study; PT: pelvic tilt, PI: pelvic incidence, SS: sacral slope. (Reprinted from Mac-Thiong et al. [6]).

Subjects of our study were 98 healthy students (50 boys and 48 girls) aged 8–19 years, who live in one of the central parts of Tehran. The study was approved by the ethical committee of our university. Goals and design of the study in addition to X-ray exposure were fully explained to them, and those children and parents who accepted the principles of study were recruited. Demographic data, history of present and past diseases, height (cm), and weight (kg) of all children were recorded. Each subject was examined by an orthopedic surgeon (3rd author). Children with more than 1 cm difference in their legs' length, history of trauma, present or past pelvic or spinal pain, disorder or abnormality, deformity proven via Adam's test, or signs of hip disorder were not included. Entirely 106 subjects had these inclusion criteria. A long cassette (30 cm in 90 cm) was chosen; children were asked to place their right side closely to the cassette in relaxed standing position, with their shoulders being 90-degree flexed and elbows fully flexed so that their fingers touched their ipsilateral shoulder. X-ray source was placed at 120 cm distance from the cassette. If femoral head or 7th cervical vertebra was not clearly seen in the radiograph (eight subjects), the subject was excluded. Eight spinopelvic parameters were measured twice by 2 orthopedic spine surgeons on each radiograph of 98 subjects separately. None of them were aware of the other surgeon's measurements. Recorded values of each surgeon for each radiograph were compared, and in case of any inconsistency, the aforesaid values were recalculated by a 3rd orthopedic spine surgeon (4th author). Assessed landmarks were superior end plates of T1, L1, S1, center of C7 body, anterosuperior of T1, L1, anteroinferior of T12, L5, center of sacral plate, and center of femoral heads. If two femoral heads were seen, the midpoint of the connecting line was selected. As shown in Figures 1, 2, and 3, parameters measured were thoracic kyphosis (T1–T12), lumbar lordosis (L1–L5), thoracic tilt (TT), lumbar tilt (LT), pelvic tilt (PT), pelvic incidence, sacral slope (SS), and sagittal vertical axis offset (SVA). Pelvic, lumbar, and thoracic tilts were assumed positive if directed forwards and negative if directed backwards. Thoracic kyphosis (TK) is the angle between lines drawn from the T1 superior end plate and T12 inferior end plate. Lumbar lordosis (LL) is the angle between lines drawn from L1 superior end plate and L5 inferior end plate. Sagittal vertical axis offset is the distance between the posterosuperior point of the sacral plate and the plumb line drawn from C7.

Thoracic tilt (TT) is the angle between the vertical line and line drawn at the anterosuperior point of T1 body and anteroinferior point of T12 body. Lumbar tilt (LT) is the angle between the vertical line and a line drawn from anterosuperior point of T1 body and anteroinferior point of T12 body (as shown in Figure 2).

SS is defined as the angle between horizontal line and superior end plate of sacrum. PI is defined as an angle subtended by line drawn center of the femoral head to the midpoint of the sacral end plate and a line perpendicular to the center of the sacral end plate. PT is defined as the angle between the vertical line and the line joining the middle of the sacral end plate and the hip axis (as shown in Figure 3).

Data was reported in mean ± SD ranges; Pearson's test was used to determine relation between parameters. Linear correlation was performed to determine the relation between PI and LL.  *P*  values less than 0.05 were considered significant. Data analysis was done with SPSS v.20.

## 3. Results 

Ninety-eight subjects, 48 girls (49%) and 50 boys (51%), with mean age of  13.6 ± 2.9  years (range: 8−19) were evaluated. Mean height and weight of children were  153.6 ± 15.6 cm and  49.9 ± 13.1 kgs.

Correlation matrix between dependent and independent parameters using Pearson's correlation and their related  *P*  values are shown in Table 2.

Thoracic kyphosis was positively related to lumbar lordosis which means lumbar lordosis would increase as thoracic kyphosis increases. LT had linear positive relation with TK. Besides PI was significantly related to LL. This relationship was positive. However, PI shows significant inverse relation with LT and was not related to TT.

## 4. Discussion 

**Figure 4:**
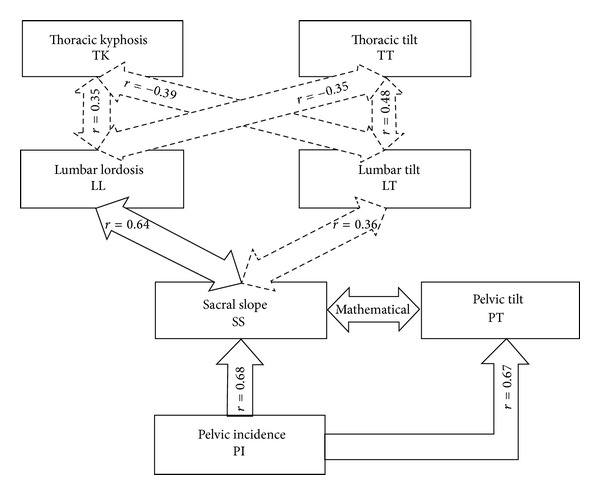
Statistical significant correlations between spinopelvic parameters introduced by Berthonnaud et al. [24] and modified by Mac-Thiong et al. [6].

Normal ranges of sagittal spinal parameters are incumbent for pre- and intraoperation planning of spinal fusion surgeries [6] to minimize energy consumption for maintaining balance [24] and to decrease the probability of junctional kyphosis [25]. This becomes more important especially when fusion expands to lower segments of the spine [26]. We launched this study on the basis that ethnicity may influence the normal ranges of these parameters. According to Table 1 it is clear that some of these parameters such as TK and LL have wide ranges whereas the tilts and spinopelvic parameters have more limited ranges. So it can be concluded that parameters with narrow spectrum may be a better tool to predicate normal or abnormal standing posture. Although PI has a wide normal range it is a constant amount for each person [27]. Mean PI in this study was  45.37 ± 10.7  (range: 4–70) which is in accordance with Descamps et al. [28] study in which the mean age of participants was close to the same value in our study (13.5 versus 12.6 years old). However Mac-Thiong et al. [5] who investigated children with a mean age of 12.0 years reported normal mean PI equal to 48.4, which is 3 degrees more than our population. LL and TK4 normal ranges of our study were 2–67 and 6–73 degrees, respectively. As mentioned before, normal sagittal spinal parameters have been less described in Asian population in comparison to western populations. Korean children have less LL, SS, and PI than Caucasian children as Lee et al. [19] reported. Takemitsu et al. [23] evaluated 13- to 16-year-old Japanese boys and girls and reported a mean TK 41° which is not compatible with values obtained in Caucasians [29–31]. LL, SS, and TK in our study is in accordance with Lee et al. and Takemitsu et al. results.  Table 3 shows the mean LL, TK, and PI in this study and some previous ones. These data demonstrate that in current study population the mentioned parameters are less than Caucasians. These 8 parameters can be categorized in 3 groups as Berthonnaud [24] and Mac-Thiong et al. [6] have shown: (a) morphologic parameters including PI, (b) segmental shape parameters such as LL and TK, and (c) orientation parameters such as tilt, SS, and SB. Considering global balance importance it seems that using PI or group (b) parameters in order to determine spinal abnormalities is not suitable enough; first PI is exclusive to each person and does not change with changes in position or with deformities. Second, group (b) parameters have a wide normal range [29, 32, 33] and it is difficult to exactly determine normal range of shape parameters. On the other hand group (c) parameters which encounter limited normal values are closely related to global balance; hence the latter parameters are better to determine spinal abnormalities than the former ones. In other words, as Stagnara et al. [34] suggested, predicating the term “abnormal” to amount of lordosis or kyphosis observed in any segment of spine which is not within the aforementioned ranges seems to be false, since there are various values of kyphosis and lordosis in the normal population which ultimately reach proper balance. So it is obvious that segmental elements are less to be counted upon than the overall balance. Regardless of sagittal spinal parameters, the relationship with each other is another matter of importance [27]. Pelvic orientation is clearly related to spinal sagittal posture [6]; once lordosis increases, SS is augmented. PI is also an important morphologic parameter in this study. It is the summation of 2 position-dependent parameters: SS and PT. In standing position, pelvic morphology, which is indicated by PI, is the main determinant of spatial orientation [27]. PI = SS + PT so if the PI increases SS, PT, or both increase as well. Berthonnaud et al. [24] published an algorithm in 2005 which is of great interest (Figure 4).

Mac-Thiong et al. [6] found that PI and LL have the most evident clinical relationship which should be considered in preoperative planning of spinal surgical operations. We also found a strong positive relationship between PI and LL  *r* = 0.56,  *P*  value < 0.001. Figure 4 confirms this linear relation as well. Other researchers have also emphasized the determinant role of PI in sagittal curves' shapes [12, 13, 24, 32, 35, 36]. PI plays its role via significant correlation with SS (*r* = 0.62,  *P*  value < 0.001), as similarly shown in the algorithm, and tight relationship with LL.

There are some differences between relations in Figure 4 and relations obtained from this study. According to Table 2 some of the relations are applicable to results of the algorithm: relations between PI and SS, LL and SS (*r* = 0.57,  *P*  value < 0.001), and LL and TK (*r* = 0.34,  *P*  value = 0.001) unlike TT which was not significantly related to LT and LL. In addition LT was positively related to TK (*r* = 0.47,  *P*  value < 0.001) and negatively related to SS (*r* = − 0.32, *P*  value = 0.001), in linear correlation the following equation was obtained:

(1) $$\begin{array}{c} \text{LL}=0.5555\times \text{PI}+10.38. \end{array}$$

This equation is relatively similar to Mcthiong's equation LL = 0.5919 × PI + 29.461 [27], particularly the constant. Thus we suggest using PI in preoperative planning of patients with spinal deformity instead of applying a certain normal value of lordosis or kyphosis. Estimating expected LL by calculating PI before operation seems reasonable, especially when taking into account that PI has a linear relation with LL. It should be kept in mind that standard sampling and large sample size are the prerequisites of estimating normal values of any population. So sampling is one of the limitations of this study. In this study we evaluated the sagittal spinal parameters below C7, whereas cervical lordosis which could influence the global balance of the spine [6] was not studied. The authors are investigating other sagittal and spinopelvic parameters on a larger population including cervical lordosis and the results will be published soon.

## 5. Conclusion 

Preoperation planning for spinal fusion surgeries applying PI seems applicable. Predicating “abnormal” to lordosis and kyphosis values alone without considering global sagittal balance is incorrect. Mean of SS and TK in our population was slightly less than that in Caucasians.

## Figures and Tables

**Table 1:** Descriptive values of parameters.

Parameter^†^	Minimum	Maximum	Mean	Standard deviation
TK	6	73	47.47	12.7
LL	2	67	39.62	12.4
TT	0	21	7.08	4.9
LT	0	24	12.09	5.9
PT	0	27	10.32	6.5
PI	4	70	45.37	10.7
SS	13	55	35.37	8.1

^†^All values in degrees.

**Table 2:** Correlation matrix between parameters and their related *P* values.

		Weight	TK	LL	TT	LT	PT	PI	SS	SVA
Correlation	Height	0804**	0.217*	−0.038	−0.088	0.216*	0.068	−0.028	−0.134	−0.112
*P* value		0.0	0.032	0.709	0.388	0.033	0.507	0.784	0.188	0.274
Correlation	Weight		0.205	0.048	−0.135	0.177	0.157	0.084	−0.106	−0.001
*P* value			0.043	0.64	0.184	0.081	0.122	0.41	0.299	0.994
Correlation	TK			0.34**	−0.18	0.47**	−0.15	0.004	0.116	−0.106
*P* value				0.001	0.07	0.0	0.156	0.97	0.254	0.299
Correlation	LL				0.065	−0.087	0.117	0.56**	0.57**	−0.24*
*P* value					0.524	0.395	0.252	0.0	0.0	0.016
Correlation	TT					0.065	0.202*	0.146	−0.028	−0.53**
*P* value						0.522	0.046	0.148	0.788	0.0
Correlation	LT						−0.26*	−0.38**	−0.32**	−0.51**
*P* value							0.011	0.0	0.001	0.0
Correlation	PT							−0.57**	−0.162	−0098
*P* value								0.0	0.111	0.338
Correlation	PI								0.62**	0.055
*P* value									0.0	0.593

*Significant at 0.01; **Significant at 0.05.

**Table 3:** Mean age, pelvic incidence, lumbar lordosis, and thoracic kyphosis of some previous studies on white population and current study.

TK (degrees)	LL (degrees)	PI (degrees)	Age (years)	Year published	Authors
Mean ± SD					
43.0 ± 10.4	48.5 ± 12.4	48.4 ± 11.2	4–18	2004	Mc-Thiong et al. [5]
43.0 ± 10.4	48.5 ± 12.4	48.4 ± 11.2	5–19	2007	Mc-Thiong et al. [6]
38 ± 10	64 ± 10	Not available	5–21	1998	Vedantam et al. [9]
37.1 ± 9.9	39.6 ± 12.4	45.3 ± 10.7	8–19	2013	Current study
